# Data on upstream segment of a hydrocarbon supply chain in Saudi Arabia

**DOI:** 10.1016/j.dib.2019.104804

**Published:** 2019-11-14

**Authors:** Ahmed M. Attia, Ahmed M. Ghaithan, Salih O. Duffuaa

**Affiliations:** a Department of Systems Engineering, King Fahd University of Petroleum & Minerals, Dhahran, Saudi Arabia; b Department of Construction Engineering & Management, King Fahd University of Petroleum & Minerals, Dhahran, Saudi Arabia

**Keywords:** Hydrocarbon supply chain, Tactical planning, Capacity planning, Decision making, Supply chain optimization

## Abstract

This article provides data related to different activities in the hydrocarbon supply chain (HCSC). HCSC structures from Crude oil and natural gas supply chains. There are three main sectors in the HCSC related to activities in the production areas, processing plants, and demand terminals. The considered activities comprise the upstream sector of the crude oil and all sectors of the natural gas. The data were collected from Saudi Arabia, considering the main production and processing plants. The provided data are useful in tactical planning of crude oil, natural gas, and natural gas products of the HCSC. These data support the research article entitled “A Multi-Objective Optimization Model for Tactical Planning of Upstream Oil & Gas Supply Chains” [1].

**Table:** Specifications Table

Subject	Decision Sciences
Specific subject area	Management Science, Operations Research, and Petroleum Supply Chain
Type of data	Tables (Excel files)
How data were acquired	Published data and distance measurement based on satellite images
Data format	Raw, processed and analyzed data
Parameters for data collection	Parameters are stable over the planning horizon, such as, price, demand and yield.
Description of data collection	Publications from Saudi Arabia General Authority for Statistics, Saudi ARAMCO, OPEC, and literature.
Data source location	Saudi Arabia
Data accessibility	Direct URL to data: https://www.dropbox.com/sh/7x5dmwqdg947az4/AAATQuFh0bFvzdquLN_NcjVaa?dl=0
Related research article	A.M. Attia, A.M. Ghaithan, S.O. Duffuaa, A multi-objective optimization model for tactical planning of upstream oil & gas supply chains, Comput. Chem. Eng. 128 (2019) 216–227.

**Table:** 

**Value of the Data** • The primary goal of publishing these data is to help other researchers to validate their models in the area of HCSC planning and management. • These data allow other researchers to tackle the problem of HCSC planning in a more integrated form regarding products and activities. • The provided data motivates the academic sector to work more in developing better optimization models for HCSC planning. • The published data can assist the decision-makers in studying the trade-off among different feasible decisions. Besides, taking decisions related to production planning, inventory management, and logistics. • These data can be used to generate more scenarios to study HCSC under uncertainty.

## 1 Data

Data related to the activities on the upstream sector of the crude oil and all sectors of the natural gas represents the projected part of the HCSC. Figure 1 presented in Ref. [1] depicts a schematic representation of the activities of interest. Related data to these activities were collected from Saudi Arabia HCSC considering a relevant level of detail and main production and processing plants.

The provided data can be classified as four groups, as follows:

1. Stream composition and yield at each plant:
  • Gas-oil ratio (GOR) corresponding to crude oil type for different reservoir streams.
  • Crude oil composition; yield of main components (e.g., natural gas, H2S) at each entity.
  • Natural gas composition, for instance, the yield of CO_2_, H_2_S, methane, and ethane.
2. The demand for crude oil and gas by-products by local customer, local industry, and international customer and the corresponding selling prices.
3. The capacity of each entity, the capacity of routes connecting the entities, and the transportation modes utilized through these routes.
4. Cost elements: production and processing costs at each entity, and transportation costs.

## 2 Experimental design, materials, and methods

This article includes quantitative data collected from puplished reports, official and government websites of Saudi Arabia, and Google Maps web. Cost of transportation via the pipelines has been estimated based on that it costs US$0.6 to transport and pump an oil barrel from Ras Tanura to Yanbu. It is estimated that the pipeline transportation cost per barrel is SR0.0016466/Km (US$l = SR3.75). To estimate the transshipment cost of each barrel, the Google Maps web [2] was used to measure the distance between any two entities of HCSC. Then transportation cost is determined by multiplying the distance times the cost of shipping a barrel per KM. It is estimated that the pipeline transportation cost per barrel is SR0.00165/Km (US$l = SR3.75).

The local demand for crude oil and gas products have been estimated based on the population size of Saudi Arabia as per 2016 (32.28 Million) [3]. The local demand then was estimated for each region by dividing the total domestic consumption per number of citizens of each region. The international demand was obtained from [4]. Then the demand of each international terminal is calculated by dividing the total international consumption per the terminal capacity. The penalty cost of producing more than the demand was estimated to equal the cost of storing. While the penalty cost of producing below the demand was estimated to equal the cost of satisfying demand from international markets including.

The gas oil ratio (GOR) and yiels of by-products were collected from puplished article [5] Plants capacities were collected from the Annual Saudi Aramco report of 2016 called [6]. International prices of crude oil, natural gas, and by-products were collected from the website of U.S. Energy Information Administration (EIA) [7, 8]. Local prices were collected from the General Authority for Statistics agency in Saudi Arabia [9].
